# One million more midwives: Why the world needs them now

**DOI:** 10.18332/ejm/214527

**Published:** 2025-12-12

**Authors:** International Confederation of Midwives (ICM)

**Affiliations:** 1 International Confederation of Midwives (ICM), The Hague, The Netherlands

**Keywords:** midwives, global petition, midwifery workforce, campaign

In October 2025, the ICM launched *One Million More Midwives*, a global petition to grow, support, and sustain the midwifery workforce. The campaign aims to collect one million signatures – one for every midwife the world urgently needs – and to draw attention to a workforce gap with serious consequences for women, newborns, and families worldwide, including in Europe.

## A preventable toll on women and newborns

Every two minutes, a woman dies from causes related to pregnancy or childbirth. Every 17 seconds, a baby is lost before birth. Each year, 2.3 million newborns die within the first 28 days of life. Many of these deaths are preventable with timely, respectful, evidence-based care^1^.

At the same time, cesarean birth rates are nearing 30% worldwide, roughly double the World Health Organization’s recommended rate, often without improving outcomes^2^. Many women experience obstetric violence^3^, millions still have unmet family planning needs, and almost half of abortions are unsafe^4^. These figures show health systems failing to meet people’s needs and to prioritize sexual, reproductive, maternal, newborn and adolescent health.

Midwives are central to the solution. Evidence shows that midwives can provide 90% of essential sexual, reproductive, maternal, newborn, and adolescent health (SRMNAH) services^5^. But the world is one million midwives short. If we had universal coverage of midwife-delivered interventions by 2035, we could prevent 67% of maternal deaths, 64% of newborn deaths, and 65% of stillbirths – saving more than 4.3 million lives^6^.

## Where does ‘one million more midwives’ come from?

The figure behind this campaign has a clear evidence base. In 2021, the *State of the World’s Midwifery* (SoWMy) report, developed by UNFPA, WHO and ICM, estimated a global shortage of 0.9 million midwives^7^. These were pre-COVID-19 figures, based on data collected before the pandemic that disrupted health systems, increased burnout, and pushed many health workers out of the workforce.

Since 2021, population growth and rising health needs have increased pressure on health systems, while many countries struggle to retain and replace midwives due to poor working conditions, lack of recognition, unsafe workplaces, and limited career opportunities.

Recognizing that the 0.9 million figure no longer reflects today’s reality, ICM has commissioned new research to update the global estimate of the midwifery workforce gap.

This research pinpoints a global shortage of one million midwives. The full analysis is currently undergoing peer review and is expected to be published in early 2026. The *One Million More Midwives* campaign is grounded in this new evidence and is designed to send a strong message to decision-makers that investment to grow, support and sustain the global midwifery workforce cannot wait.

## A global petition with local impact

The petition is hosted at millionmore.org and available in seven languages. Anyone can sign it – midwives, other health professionals, women and families, students, advocates, and the general public. The website also offers resources to help people share the campaign widely.

The petition will remain open until the final day of the ICM Triennial Congress in Lisbon in June 2026. There, ICM will present the signatures to global leaders as a visible, collective demand to:

- Increase the number of educated, regulated, and wellsupported midwives
- Improve working conditions, safety, and fair pay for midwives
- Integrate midwives into policy, leadership, and decision-making spaces
- Ensure midwives can work to their full scope of practice, in collaboration with other health professionals

After the petition closes, ICM will share with each member association the number of signatures collected from their country. Associations will also receive tailored advocacy tools to help them use these results in national dialogue with governments, professional bodies, and other stakeholders.

## Why this matters for European midwives

Although the largest midwifery gaps are in low- and middle-income countries, workforce shortages and pressures are also a reality in Europe. Ageing workforces, unsafe workloads, burnout, and insufficient investment in education and retention, affect midwives’ ability to provide the quality, continuous, woman-centered care they are trained for.

One million more midwives worldwide is not only a global target – it is a call to every country, including those in Europe, to examine whether midwives are valued, protected, and enabled to do their work. For midwives in Europe, signing and sharing the petition is a way to stand in solidarity with colleagues globally while also drawing attention to the gaps and challenges within their own systems.

## A call to action

The message of *One Million More* is simple: if we want fewer preventable deaths, less obstetric violence, and more rights-based, high-quality care, the world needs more midwives – and better support for those already in post.

ICM invites all midwives, health professionals, professional associations, and the general public to visit millionmore.org, sign the petition, and help spread the word. Together, these signatures will send a powerful message to invest in, support, and respect midwives worldwide.

## Data Availability

Data sharing is not applicable to this article as no new data were created.
